# Antimicrobial use in paediatric patients in a teaching hospital in Ethiopia

**DOI:** 10.1371/journal.pone.0173290

**Published:** 2017-03-06

**Authors:** Hafte Kahsay Kebede, Hailay Abrha Gesesew, Tewodros Eyob Woldehaimanot, Kabaye Kumela Goro

**Affiliations:** 1 Filtu Hospital, Somali, Ethiopia; 2 Department of Epidemiology, College of Health Sciences, Jimma University, Jimma, Ethiopia; 3 Discipline of Public Health, Faculty of Medicine, Nursing, and Health Sciences, Flinders University, Adelaide, Australia; 4 Department of Clinical Pharmacy, College of Health Sciences, Jimma University, Jimma, Ethiopia; University of Nottingham, UNITED KINGDOM

## Abstract

**Background:**

Antibiotics use in in children are different from adults due to a lack of data on pharmacokinetics, pharmacodynamics, efficacy and safety of drugs, different physiological spectrum, pediatrics populations being vulnerable to the majority of the illnesses, and the adverse effect of their irrational use is more serious. However, antibiotic use is not explored much in a paediatric population. The current study focused on antibiotic use among pediatric population using data from a tertiary hospital in Ethiopia.

**Methods:**

A retrospective cross-sectional study collated data from 614 pediatrics patients admitted in pediatrics ward at Jimma University Teaching Hospital, Southwest Ethiopia. Descriptive analyses were performed to describe the type and pattern of antibiotics. The number of prescriptions per a patient was also compared with the WHO standard. Data analysis was carried out using SPSS version 20 for mackintosh.

**Results:**

Antimicrobials were prescribed for 407(86.4%) patients of which 85.9% were in the form of injectables. A total of 1241 (90%) medicines were administered parenterally followed by oral 110 (8%). The maximum number of medicines per prescription was eight for all types of drugs in general, and five for antimicrobials in particular. All antimicrobials were prescribed empirically without any microbiological evidence. Pneumonia, sepsis and meningitis were the main reasons for antimicrobial use in the ward. Out of the total of 812 antibiotics prescribed; Penicillin G crystalline was the most (20%) frequently prescribed, followed by gentamicin (19%) and ampicillin (16).

**Conclusions:**

Majority of the prescribed antibiotics were antimicrobials, and was in the form of injectables. Antimicrobials were over prescribed and the number of drugs per prescription was also far from WHO recommendation. Strict prescribing standard guidelines and treatment habits should be developed in the country, to prevent antimicrobial resistance.

## Background

The invention of antimicrobials emerged as a transformational turning point in the reduction of the burden of communicable disease in the 20^th^ century[1]. Antimicrobials are among the most widely prescribed therapeutic agents across the world [2–4]. The use of antibiotics among children is different from sulta due to a number of reasons but not limited to: (i) a lack of data on pharmacokinetics, pharmacodynamics, efficacy and safety of drugs[5], (ii) different physiological spectrum among different age groups -preterm neonates, full term neonates, infants and toddlers, and older children and adolescents [5], (iii) pediatrics populations being vulnerable to the majority of the illnesses[6], and (iv) the adverse effect of irrational use of antimicrobials being more serious among children than adults[7].

If antibiotics sued inappropriately, it leads to the emergence and worrying national and global trends of antimicrobial resistance[8–10]. According to the WHO, the rational medicine use included the appropriate use of medicine, in the proper dose, for an adequate period of time, and at the lowest cost to the individuals and their community [11]. Globally, irrational drug use is a serious problem[12], with WHO estimating that more than half of all medicines are being inappropriately prescribed, dispensed or sold [12]. The prevalence rates of inappropriate antimicrobial use of 37, 47, and 8% in India[13], Turkey[14] and Israel[15] respectively have been reported. Further studies in Africa including from Nigeria[16] and Ethiopia[7] have also reported high magnitude of inappropriate use of medicines. It is well known that prescribing against clinical guidelines, inappropriate self-medication, and improper use of medicines such as overuse, underuse and misuse can be described as inappropriate use of drugs [17,12], and can lead to antimicrobial resistance [9]. In addition, inappropriate drug use leads to treatment failure, high incidence of toxicities and waste of financial resources[18].

In Ethiopia, it has been stated that there is a high rate (55%) of prescriptions that comprises one or more antimicrobials[19]. Studies from Northwest[20] and Southwest[21] of the country documented that antimicrobials accounted for 60 and 26% of all prescriptions respectively. Many pediatrics physicians in Ethiopia habitually prescribe antimicrobials at times can be rational or irrational[22]. In addition, 50% of Ethiopian hospitals’ budget is allocated to antimicrobials[23]. Nevertheless, there is limited number of studies that have described antimicrobial use among children. We assessed antibiotic use among pediatric population using data from a pediatrics ward at Jimma University Teaching Hospital (JUTH), Southwest Ethiopia.

## Material and methods

### Study design, settings and participants

A retrospective cross-sectional study was carried out in a pediatrics ward at JUTH, Southwest Ethiopia from March 20 up to April 20, 2014. The hospital serves a catchment area of three million people from rural, urban and semi-urban areas. JUTH pediatrics ward serves children who come from out patient departments (OPD) and other wards such as surgical ward. Records of all pediatrics (children ≤ 14 years of old) who were admitted in JUTH were the target population. Incomplete records were excluded from the analysis.

### Sampling procedure

The required sample size was calculated via OpenEpi software using single population proportion calculation formula based on the following assumptions: 58% prevalence rate of antimicrobial use[24], 95% confidence level, 4% margin of error and 5% incomplete files. The total calculated sample of records yielded 614. The records were selected randomly using sampling frame from pediatrics records between January 2012 and February 2014. All prescribed antimicrobials of the randomly selected records were evaluated.

### Variables in the study

The variables the study included sex, age, co-morbidity index, duration of hospital stay, number of prescribed antimicrobials, purpose of antimicrobial order, surgical procedure done, antimicrobial per encounter, concomitantly used medications and antimicrobial use. Purpose of antimicrobial order was assessed if the antimicrobial was prescribed for therapy, prophylaxis, both or else. Number of diagnosis is the number of diseases explicitly recorded as diagnosis by physician.

### Data collection process and statistical analysis

Data were extracted from records of pediatrics patients who were admitted at JUTH pediatrics ward from January 2012 to February 2014. A data extraction checklist was prepared in English. Four hospital pharmacists, who had been trained on how to extract and use the instruments, extracted the data. To ensure the quality of data, the principal investigator and a supervisor checked the data extraction tools for completeness and consistency daily. Descriptive statistics included mean, median, standard deviations, and range values for continuous data; percentage, frequency tables and graphs for categorical data. The analysis was conducted in Statistical Package for the Social Sciences (SPSS) version 20 for mackintosh.

### Ethical considerations

The study was approved by Institutional Review Board (IRB) of College of Health Sciences at Jimma University, Southwest Ethiopia. Permission for the study to be conducted was also obtained from JUTH. Information gathered was treated as confidential and accessible only to the investigators. Data were extracted for pediatrics age patients on targeted variables. Only de-identified data were extracted.

## Results

### Demographic and clinical characteristics of study participants

Six hundred and fourteen (614) patients attending the JUTH’s pediatrics ward between January 2012 and February 2014 were initially considered eligible. Of these, 143 records were excluded since their charts were not available or incomplete. In total, 471 patients’ records were reviewed. Table 1 shows demographic and clinical characteristics of the participant who received antimicrobial prescription. More than two third of the patients received two or more antimicrobials. The proportion of males and females was similar and the over one third (35%) of the participants represented toddlers and preschool children aged 1–5 years. Compared to urban dwellers, rural settlers comprised more than half (59%) of the study participants.

**Table 1:** Demographic and clinical characteristics of study participants.

Variables)	Levels	Frequency (n = 407)
Sex	Female	175(43)
	Male	232(57)
Age	0–27 days	56(14)
	1month- 1year	137(34)
	1–5yrs	144(35)
	5–14yrs	70(17)
Residence	Urban	169 (42)
	Rural	238 (59)
Duration of hospital stay (days)	≤5	207(51)
	>5–10	86(21)
	>10	114(28)
Co-morbidity index	0	202(50)
	≥1	205(50)
Purpose of antimicrobial order	Therapy	364(89)
	Prophylaxis	31(8)
	Not documented	12(3)
Number of antimicrobials per encounter	1	131(32)
	≥2	276(68)
Surgical procedure done	Yes	48(12)
	No	359(88)
Number of patients concomitantly on other medication	0	171(42)
	≥1	236(58)

Half (51%) of the patients had a hospital stay of ≤ 5 days with median hospitalization stay of five days. About half (50%) of the participants had one or more co-morbidity index. Only one out of ten patients had history of surgical procedures.

### Pattern of antimicrobials prescription

A total of 1241 (90%) medicines were administered parenterally followed by oral 110 (8%). Of these, antimicrobials were prescribed for 407(86%) patients, and the majority (86%) of the antimicrobials were in the form of injectables. The majority of patients, 276 (68%), received more than one antimicrobials per one prescription order. The maximum number of medicines per prescription was eight for all types of drugs in general, and five for antimicrobials in particular. All antimicrobials were prescribed empirically without any microbiological evidence. While the main reason for antimicrobials prescription was for therapeutic, few were ordered for prophylaxis and, a small proportion were ordered for prophylaxis and therapy (Table 1). Out of the total drugs prescribed, 166(12%) were mono drug prescriptions.

Percentage of hospitalizations with one or more antimicrobials prescribed was 86%. The mean number of antimicrobials prescribed per hospitalization was two (SD = 0.91) while the mean number of antimicrobial injections prescribed per-prescription was 1.7 (SD = 1.1). In addition, the median duration of prescribed antimicrobial treatment was 7 (1–43) days, and the percentage of surgical inpatients who received antimicrobial prophylaxis was 100%. Four out of five (78.8%) of the prescribed antimicrobials were included in the Ethiopian EDL (Essential Drug List). The mean antimicrobial prescription in neonates was relatively higher (2.32; 95% C.I: 2.12–2.51, SD = 0.72) compared to the other age groups (Table 2). Table 3 reports the percentage of number and percentage of prescribed antibiotics in the Pediatric ward. Of the total of 812 antibiotics prescribed; penicillin G crystalline was the most (20%) frequently prescribed, followed by gentamicin (19%) and ampicillin (16).

**Table 2:** Pattern of antimicrobial use by different age groups.

Age category	Antimicrobials used (n = 407), n (%)	Mean antimicrobial prescription [mean (CI, SD)]
<1 month	56(14)	2.3 (2.1–2.5, SD = 0.72)
1month—1 year	137(34)	1.9 (1.7–2.1, SD = 0.94)
1year—5 years	144(35)	1.9 (1.8–2.1 SD = 0.95)
>5 years	70(17)	1.9 (1.8–2.2, SD = 0.84)
Total	407	2.0 (1.9–2.1, SD = 0.91)

**Table 3:** The percentage of prescribed Antibiotics in Pediatric Ward of Jimma University Teaching Hospital, Southwest Ethiopia.

Type of Antibiotics	Number of prescription, n (%)
Penicillin G	166 (20)
Gentamicin	150 (18)
Ampicillin	128 (16)
Cloxacillin	107 (13)
Chloramphenicol	103 (13)
Amoxicillin	66 (8)
Ceftriaxone	50 (6)
Tetracycline eye ointment	17 (2)
Others	25 (3)
Total	812 (100)

## Discussion

The current study provides important information on pattern of and type for antimicrobial use in Southwest Ethiopia. Compared to studies conducted in other countries such as in the Serbia (54%) [25], South Africa (75%)[26], Uganda (43.2%)[27] and Jordan (36.8%)[28], the current study revealed higher (86.4%) antimicrobial prescribing patterns in a hospital in Ethiopia. The plausible justification for these differences could be related to variations in prevalence rate of infectious disease, socio-cultural factors, awareness of antimicrobial resistance among health professionals, poor prescribing policy and physicians’ skill to diagnose common communicable diseases [29,30]. This may indicate that there is a need to build capacity for health care provision and to develop strict prescribing policy for antimicrobials. For these to be achieved, significant efforts need to be invested into developing standard treatment guidelines and enforcement of appropriate prescribing habits.

Of all prescribed antimicrobials, 86% were injectable antimicrobials. These injectable prescription patterns are higher than those found in studies conducted in Palestine (62%)[22] and Kathmandu Valley of Nepal (75%)[31]. The WHO recommends only 10% of injectables for the total prescriptions for a health setting, and these patterns are by far higher than these recommendations[32]. Several factors could explain and some of these observations including: (i) differences in availability of antimicrobials in other forms, (ii) the gap in skills among healthcare providers, (iii) and availability of standard treatment guidelines.

The number of drugs per prescription was far from the acceptable level compared with the standard (1.6–1.8) [32]. In addition, all antimicrobials were prescribed without any microbial evidence. These findings demonstrate the scarcity of knowledge and understanding regarding the consequences of inappropriate antimicrobial use among physicians who work in pediatrics ward. These findings call for development of drug policy in Ethiopia. Evaluation of healthcare providers prescription knowledge and behaviours would also need to be undertaken in order to addressed the above gaps.

The percentage of antimicrobials prescribed from EDL was 79% and this is very low compared to the Ethiopian health policy that recommends 100% of the prescribed drugs should be from EDL. We recommend that the Ethiopian Food, Medicine and Health Care Administration and Control Agency (FMHACA) should put more efforts to increase the awareness of health care providers and availability of the EDL guidelines in every health facility. In addition, the EDL guideline should be distributed to graduating health science students so that their potential towards the guideline heightens and its shortage in the health facility resolves.

The study has the following several limitations. There were significant (23%) incomplete patient charts. The small sample size nature of the retrospective study may limit the generalizability of the findings. The public hospital-based nature of the study may also not be generalized to public health centers and private health institutions due to the different mix of health professionals and availability of drugs. It has been reported that distribution of highly skilled health professionals is skewed towards private and nongovernmental organizations in the country [33]. For example, in 2006–07, 56% of specialists and 38% of general practitioners working in health facilities were outside the public sectors [34]. In addition, the retrospective nature of the current study cannot assure the cause-effect relationship of the significantly associated factors linked to inappropriate antimicrobial use.

## Conclusions

This study highlights antimicrobial prescription pattern in a pediatrics setting in Southwest Ethiopia. The study is significant because of the emerging and an increase in antimicrobial resistance. The study provides impetus to a national drive toward antimicrobial stewardship. In summary, the findings from the current study agree in many points with the findings of previous publications. Antimicrobials were over prescribed and their injectable forms were prescribed by far higher than the WHO recommendation. The number of drugs per prescription was also far from the acceptable level. In addition, the percentage of antimicrobials prescribed from essential drug list was less than from the national recommendations and expectations.

Developing effective interventions to reduce inappropriate antimicrobial prescribing will require a clear understanding of how these predictors influence antimicrobial prescribing by large scale prospective studies throughout the country. Strict prescribing policy and standard treatment guideline-appropriate prescribing habits should also be developed and enforced in Ethiopia.
